# Supplementary material for: Negative elongation factor complex enables macrophage inflammatory responses by controlling anti-inflammatory gene expression
Source: Nat Commun. 2020 May 8;11:2286. doi: 10.1038/s41467-020-16209-5 (PMC7210294; doi:10.1038/s41467-020-16209-5)
Supplement: Supplementary file 1 — Supplementary Information [file 41467_2020_16209_MOESM1_ESM.pdf]

Supplementary information for

Yu et al. “Negative elongation factor complex enables macrophage inflammatory responses by controlling anti-inflammatory gene expression”

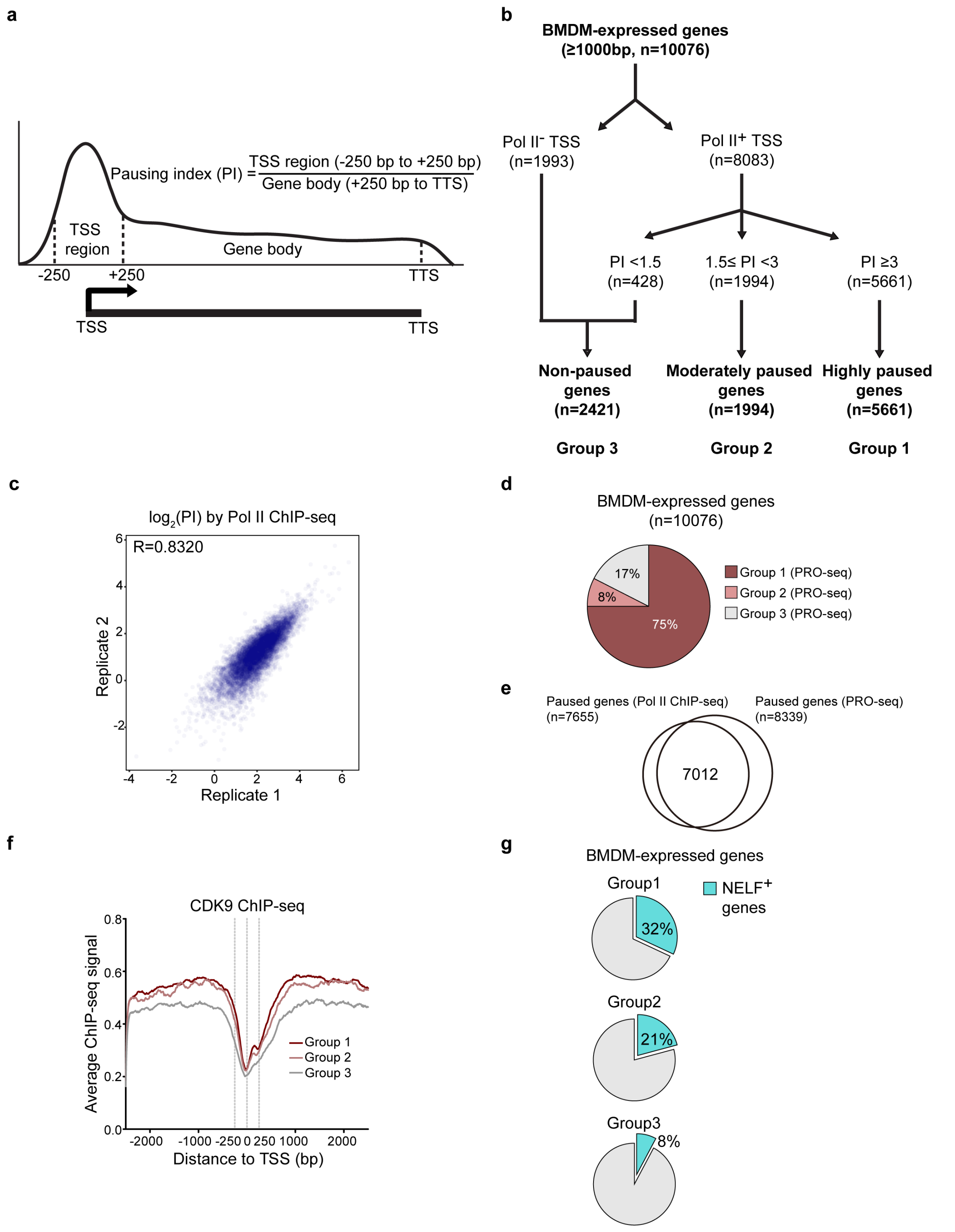

Supplementary Figure 1. BMDM-expressed genes classified by Pol II distribution patterns. (**a**) Calculation of Pol II pausing index (PI). For each gene, PI is defined as a ratio of Pol II ChIP-seq read density in the TSS region/gene body region. (**b**) A flow chart

shows the scheme of BMDM-expressed genes classified according to Pol II distribution in resting BMDM. Genes with PI ≥ 3 are defined as highly paused (group 1), and genes with 1.5 ≤ PI < 3 as moderately paused (group 2). Genes with either PI < 1.5 or TSS region lacking apparent Pol II occupancy are defined as non-paused (group 3). (**c**) A scatter plot shows the correlation of PI between two biological replicates in resting BMDM. x- and y-axes indicate $\log_2$ normalized PI in replicates 1 and 2, respectively. Correlation coefficient (R) is shown. (**d**) Pie graph shows the percentage of all BMDM-expressed genes in each group based on PRO-seq defined PI. (**e**) The overlap between Pol II ChIP-seq defined and PRO-seq defined paused BMDM-expressed genes. (**f**) Average CDK9 ChIP-seq signals around TSS regions of group 1 (brown), group 2 (red) and group 3 (grey) genes in resting BMDM. (**g**) The percentage of $NELF^+$ genes in group 1, 2 and 3 of BMDM-expressed genes.

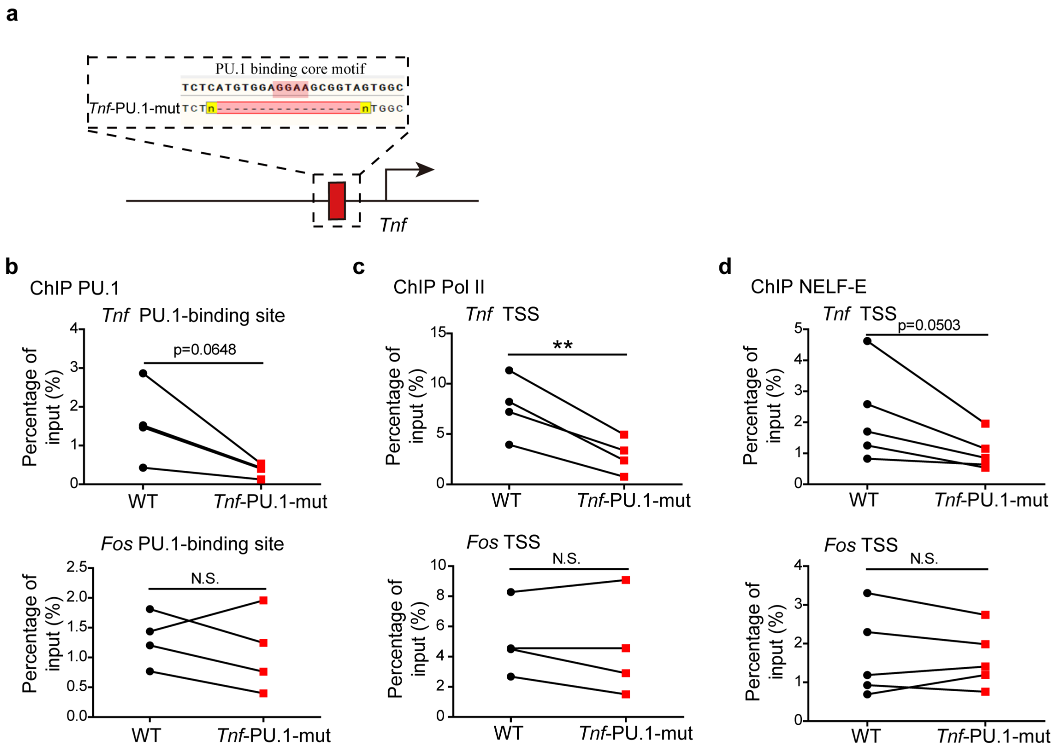

Supplementary Figure 2. Promoter-proximal PU.1 contributes to Pol II pausing. (**a**) PU.1 motif deletion in the *Tnf* promoter region (termed *Tnf*-PU.1-mut). (**b**) The occupancy of PU.1 (n=4) at the PU.1 binding site in the *Tnf* (top, *P*=0.0648) and *Fos* (bottom, *P*=0.4506) promoter region was assessed by ChIP-qPCR in WT and *Tnf*-PU.1-mut iBMDM. (**c**, **d**) The occupancy of Pol II (n=4) (**c**) and NELF-E (n=5) (**d**) in the TSS regions of *Tnf* (Pol II *P*=0.0082; NELF-E *P*=0.0503) and *Fos* (Pol II *P*=0.4388; NELF-E *P*=0.7478) was assessed by ChIP-qPCR in WT and *Tnf*-PU.1-mut iBMDM. ** *P*<0.01, N.S. *P*>0.05 by two-sided paired Student's t test. n represents biologically independent experiments. Source data are provided as a Source Data file.

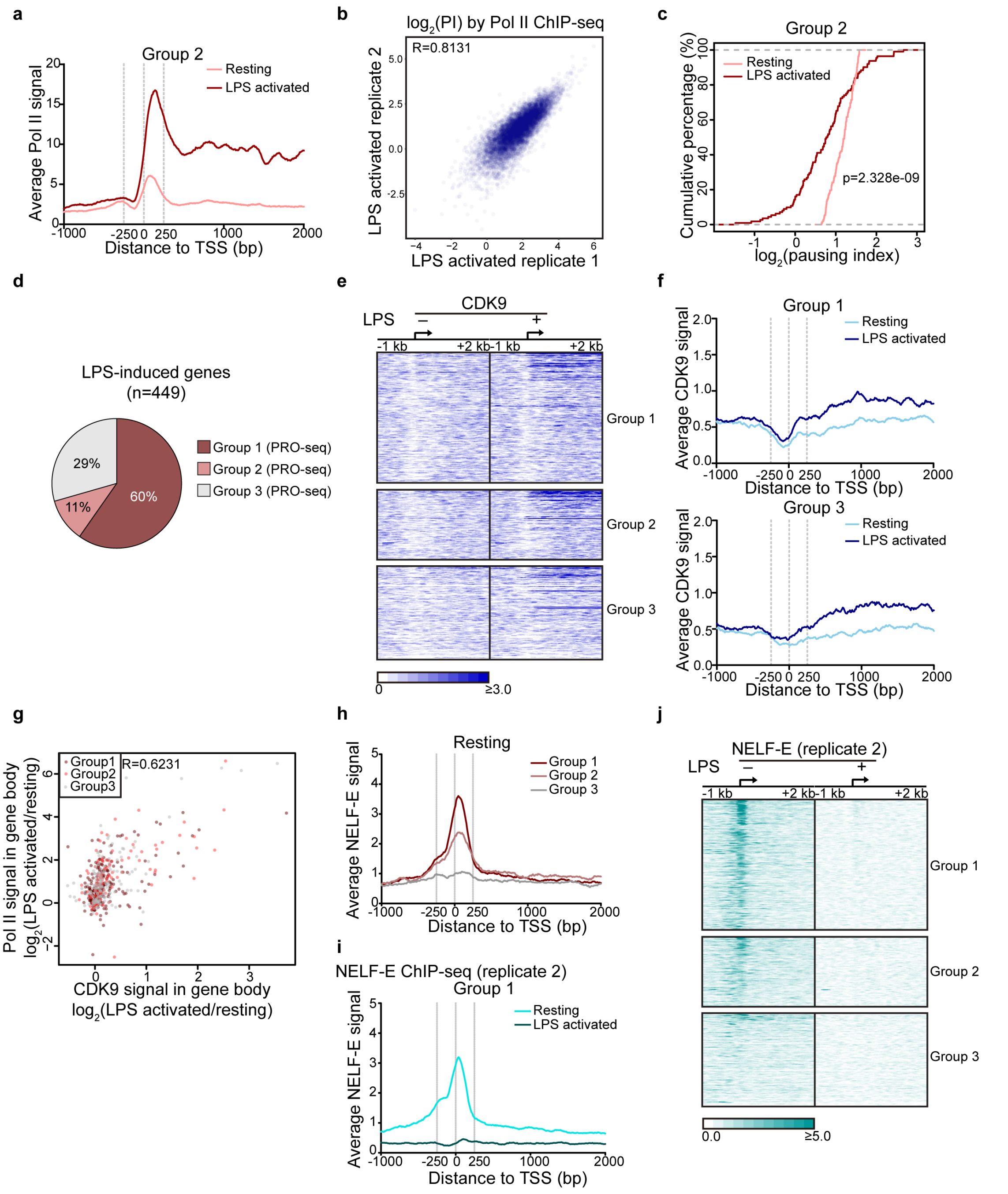

Supplementary Figure 3. NELF dismissal during Pol II pause-release is a key feature of macrophage activation. (**a**) Average Pol II ChIP-seq signals around TSS of group 2 (moderately paused) LPS-inducible genes in resting and LPS-activated (1 h) BMDM. (**b**) The correlation of PI between two biological replicates of Pol II ChIP-seq in LPS-activated

BMDM. x- and y-axes indicate log_2 normalized PI in two replicates. Correlation coefficient (R) is shown. (**c**) The ECDF plot of Pol II PI distribution of group 2 LPS-inducible genes in resting and LPS-activated (1 h) BMDM. (**d**) The percentage of LPS-induced genes in groups 1, 2, 3 re-defined by PRO-seq. (**e**) Heat map of CDK9 ChIP-seq signals around the TSS of LPS-inducible genes in resting and LPS-activated (1 h) BMDM. For each of the three groups, the rows were sorted by the decreasing Pol II ChIP-seq signal in TSS region in resting BMDM. (**f**) Average CDK9 ChIP-seq signals around TSS regions of group 1 (top) and group 3 (bottom) genes in resting and LPS-activated (1 h) BMDM. (**g**) The correlation between CDK9 ChIP-seq signals (fold changes LPS activated/resting; x-axis) and Pol II ChIP-seq signals (fold changes LPS activated/resting; y-axis) in the bodies of genes in each group. Correlation coefficient (R) is shown. (**h**) Average NELF-E ChIP-seq (replicate 1) signals around TSS regions of group 1, 2 and 3 LPS-inducible genes, as indicated, in resting BMDM. (**i**) Average NELF-E ChIP-seq (replicate 2) signals around TSS regions of group 1 genes in resting and LPS-activated (0.5 h) BMDM. (**j**) Heat map of NELF-E (replicate 2) ChIP-seq signals around the TSS of LPS-inducible genes in resting and LPS-activated (0.5 h) BMDM. For each of the three groups, the rows were sorted by the decreasing Pol II ChIP-seq signal in TSS region in resting BMDM.

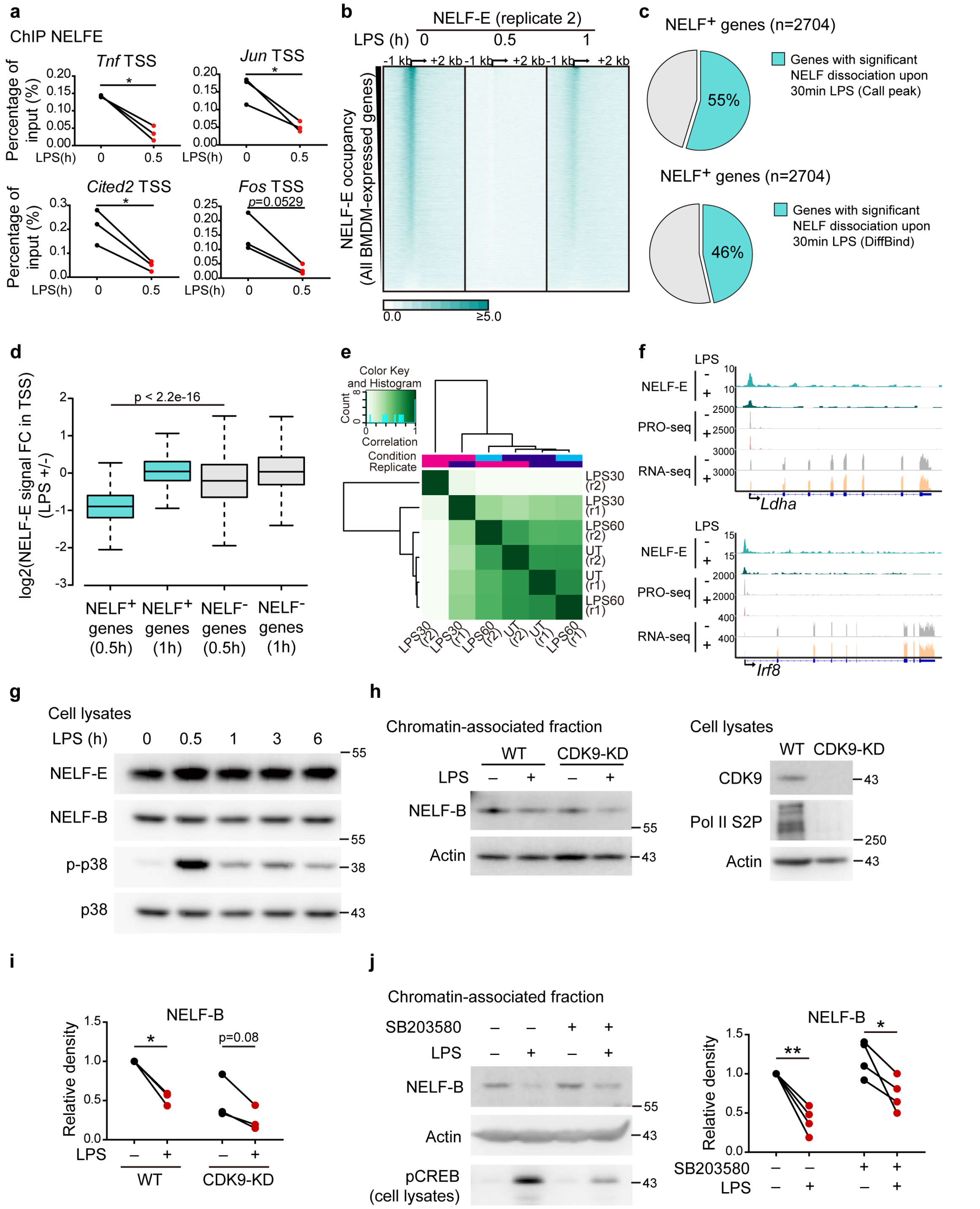

Supplementary Figure 4. NELF is globally and transiently dismissed from chromatin upon macrophage activation. (**a**) The occupancy of NELF-E (n=3) in the TSS region of *Tnf* (*P*=0.016), *Jun* (*P*=0.0434), *Cited2* (*P*=0.0325), *Fos* (*P*=0.0529) was assessed by ChIP-

qPCR in resting and LPS-activated (0.5 h) BMDM. (**b**) Heat map of NELF-E ChIP-seq (replicate 2) signals around the TSS regions of all BMDM-expressed genes in resting and LPS-activated, as indicated, BMDM. Each row indicates one gene, and the rows were sorted by the decreasing NELF-E ChIP-seq signal near TSS in resting BMDM. (**c**) Percentage of $NELF^+$ genes with significant NELF dissociation upon LPS stimulation as defined by peak calling (top) and DiffBind (bottom). (**d**) NELF-E ChIP-seq signal changes upon 0.5 h (0.5 h vs. 0 h) and 1 h (1 h vs. 0 h) LPS stimulation in TSS regions of $NELF^+$ and $NELF^-$ genes shown as boxplot. Boxes outline the data from 1st quartile to 3rd quartile, and bars indicate the boundary whose distribution is not exceeded to 1.5 fold of interquartile range extended from both 1st quartile and 3rd quartile. p-value ($NELF^+$ genes 0.5 h/$NELF^-$ genes 0.5 h $P < 2.2e^{-16}$) was calculated by two-sided Mann-Whitney U test. (**e**) Correlation heat map shows the similarity of NELF-E ChIP-seq signal in TSS regions of all unique genes among samples from two replicates upon 0.5 h and 1 h LPS stimulation. (**f**) Tracks of NELF-E ChIP-seq, PRO-seq (sense strand) and RNA-seq for representative LPS non-regulated $NELF^+$ paused genes (*Ldha* and *Irf8*) in resting (-) and LPS-activated (0.5 h, +) BMDM. (**g**) NELF-E, NELF-B, p-p38 and p38 were detected by western blotting in total cell lysates of BMDM treated with LPS for indicated time. Shown is one representative blot from three biological replicates. (**h**) Chromatin-associated NELF-B and actin were detected by western blotting in WT and CDK9-KD iBMDM with or without LPS stimulation (left). CDK9, Pol II S2P and actin were visualized in total cell lysates (right). Shown is one representative blot from three biological replicates. (**i**) For each replicate from (**h**), NELF-B bands were quantified by densitometry, normalized to internal control (actin) and expressed relative to untreated WT sample (=1) (WT LPS 0 h/WT LPS 0.5 h P=0.011; CDK9-KD LPS 0 h/CDK9-KD LPS 0.5 h P=0.0814). (**j**) Chromatin-associated NELF-B and actin were detected by western blotting in mock (DMSO) and SB203580 (20 μM) –pretreated (0.5 h) BMDM; pCREB blot from total cell lysates is used as a control for SB203580 activity. Shown is one representative result from four biological

replicates (left). For each replicate, NELF-B protein level was quantified by densitometry, normalized to internal control (actin) and expressed relative to untreated sample (=1) (right) (LPS 0 h/0.5 h P=0.0064; SB203580 pretreated LPS 0 h/LPS 0.5 h P=0.0463). * P<0.05, ** P<0.01, N.S. P >0.05 by two-sided paired Student's t test. n represents biologically independent experiments. Source data are provided as a Source Data file.

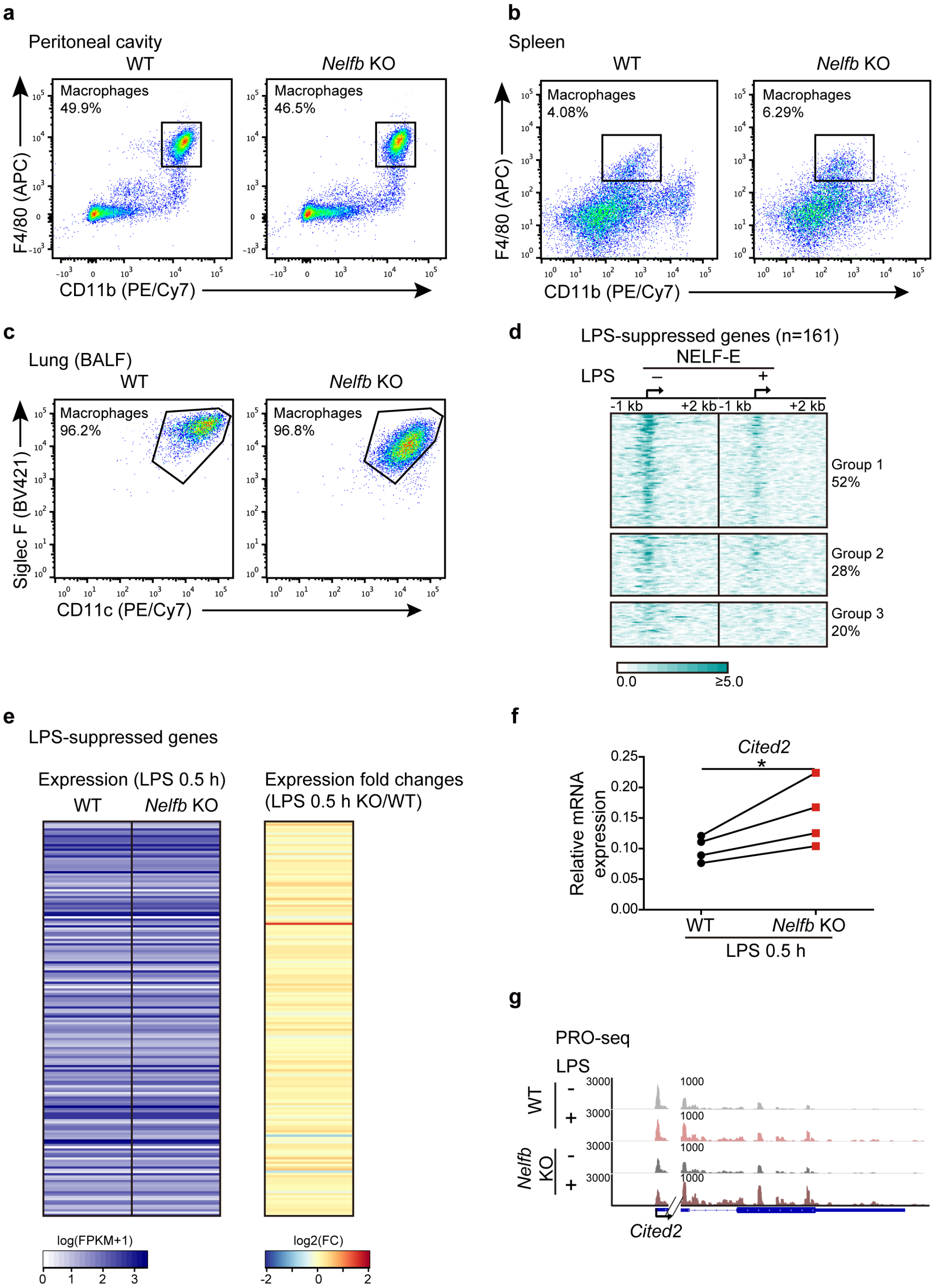

Supplementary Figure 5. NELF is involved in the transcription of LPS-suppressed gene. (**a**, **b**, **c**) FACS analysis of macrophage populations in peritoneal exudates (**a**: CD45$^+$CD11b$^+$F4/80$^+$), spleen (**b**: CD45$^+$CD11b$^+$F4/80$^+$) and lung (broncho-alveolar

lavage fluid, BALF) (**c**: $CD45^+CD11c^+$Siglec F^+) in WT and *Nelfb* KO mice. (**d**) Heat map of NELF-E ChIP-seq signals in resting and LPS-activated (0.5 h, +) BMDM around the TSS regions of group 1, 2 and 3 LPS-suppressed genes (n=161). For each group, the rows were sorted by decreasing NELF-E ChIP-seq signal in TSS regions in resting state. (**e**) Heat maps show the RNA-seq expression level (FPKM+1) in WT and *Nelfb* KO (left) and fold changes (*Nelfb* KO/WT) (right) of LPS-suppressed genes in LPS-treated (0.5 h) BMDM. (**f**) Cumulative RT-qPCR data showing expression level of *Cited2* in WT and *Nelfb* KO BMDM treated with LPS for 0.5 h (n=4 biologically independent experiments, P=0.045). * P<0.05 by two-sided paired Student's t test. (**g**) PRO-seq (sense strand) tracks for *Cited2* in WT and *Nelfb* KO BMDM cultured with (+) or without (-) LPS for 0.5 h. Source data are provided as a Source Data file.

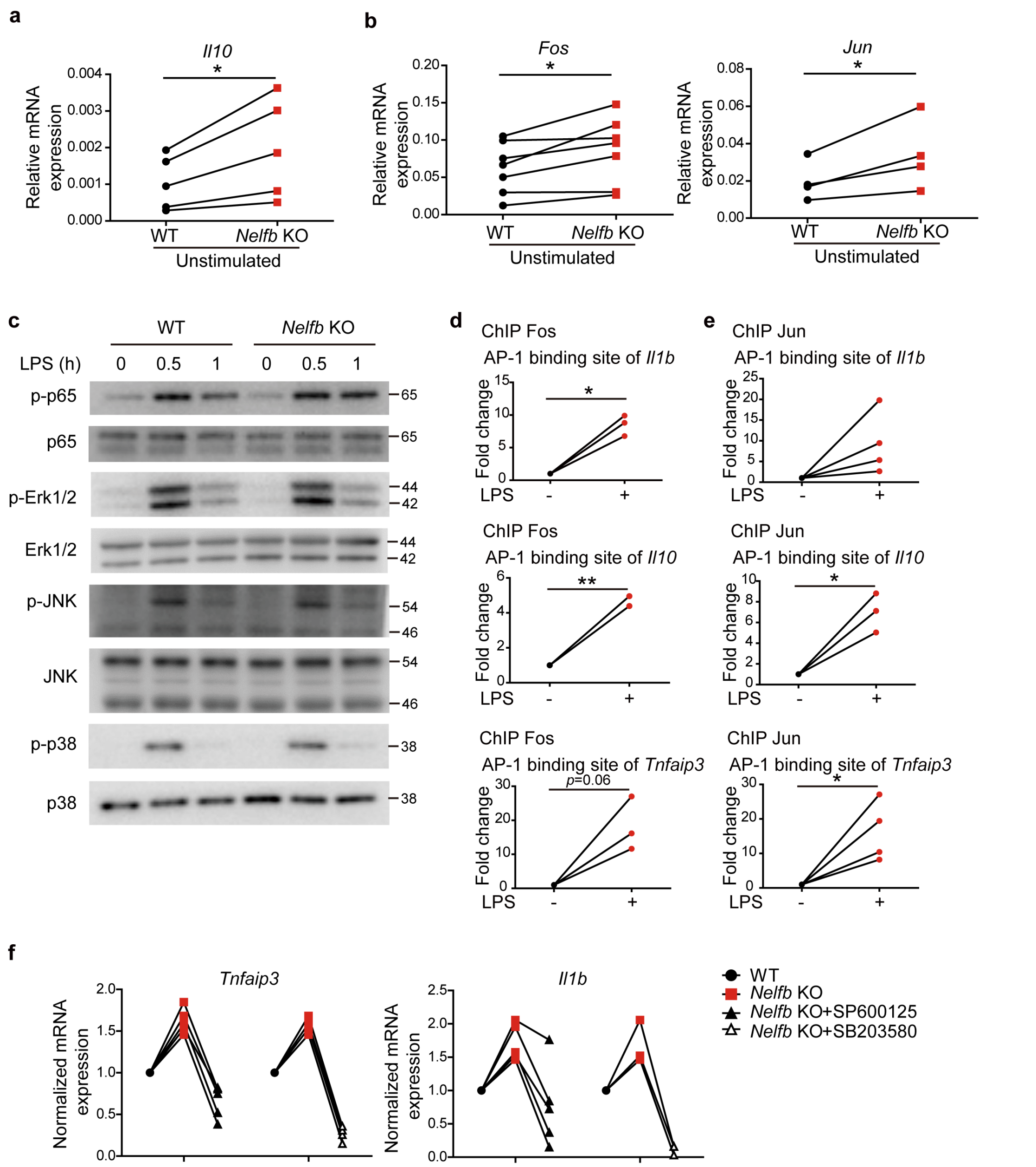

Supplementary Figure 6. NELF deficiency does not affect TLR-induced activation of canonical signaling pathways. (**a**, **b**) Cumulative RT-qPCR data showing expression level of *Il10* (n=5, *P*=0.0286), *Fos* (n=7, *P*=0.0203) and *Jun* (n=4, *P*=0.0497) in WT and *Nelfb* KO resting BMDM. (**c**) Immunoblot analysis of p-p65, p65, p-Erk1/2, Erk1/2, p-JNK, JNK, p-p38 and p38 in whole cell lysates of WT and *Nelfb* KO BMDM treated with

LPS for indicated time. Shown is one representative blot from three biological replicates. (**d**, **e**) Occupancies of Fos (*Il1b* n=3, P=0.0147; *Il10* n=3, *P*=0.0025; *Tnfaip3* n=4, *P*=0.0633) (**d**) and Jun (*Il1b* n=4, *P*=0.1146; *Il10* n=3, *P*=0.0316; *Tnfaip3* n=4, *P*=0.0389) (**e**) at the AP-1 binding site of *Il1b, Il10* and *Tnfaip3* were assessed by ChIP-qPCR in resting and LPS-activated (1 h) BMDM. (**f**) WT and *Nelfb* KO BMDM were pretreated with SP600125 (50 μM) or SB203580 (20 μM), as indicated, for 0.5 h, and *Tnfaip3* (left) and *Il1b* (right) expression following 0.5 h of LPS co-incubation was measured by RT-qPCR (SP600125-*Tnfaip3*: n=5; SP600125-*Il1b*: n=5; SB203580-*Tnfaip3*: n=4; SB203580-*Il1b*: n=3). Relative mRNA expression was normalized to the levels in WT cells that were set as 1. * $P<0.05$, ** $P<0.01$, *** $P<0.001$, N.S. $P>0.05$ by two-sided paired Student's *t* test. n represents biologically independent experiments. Source data are provided as a Source Data file.

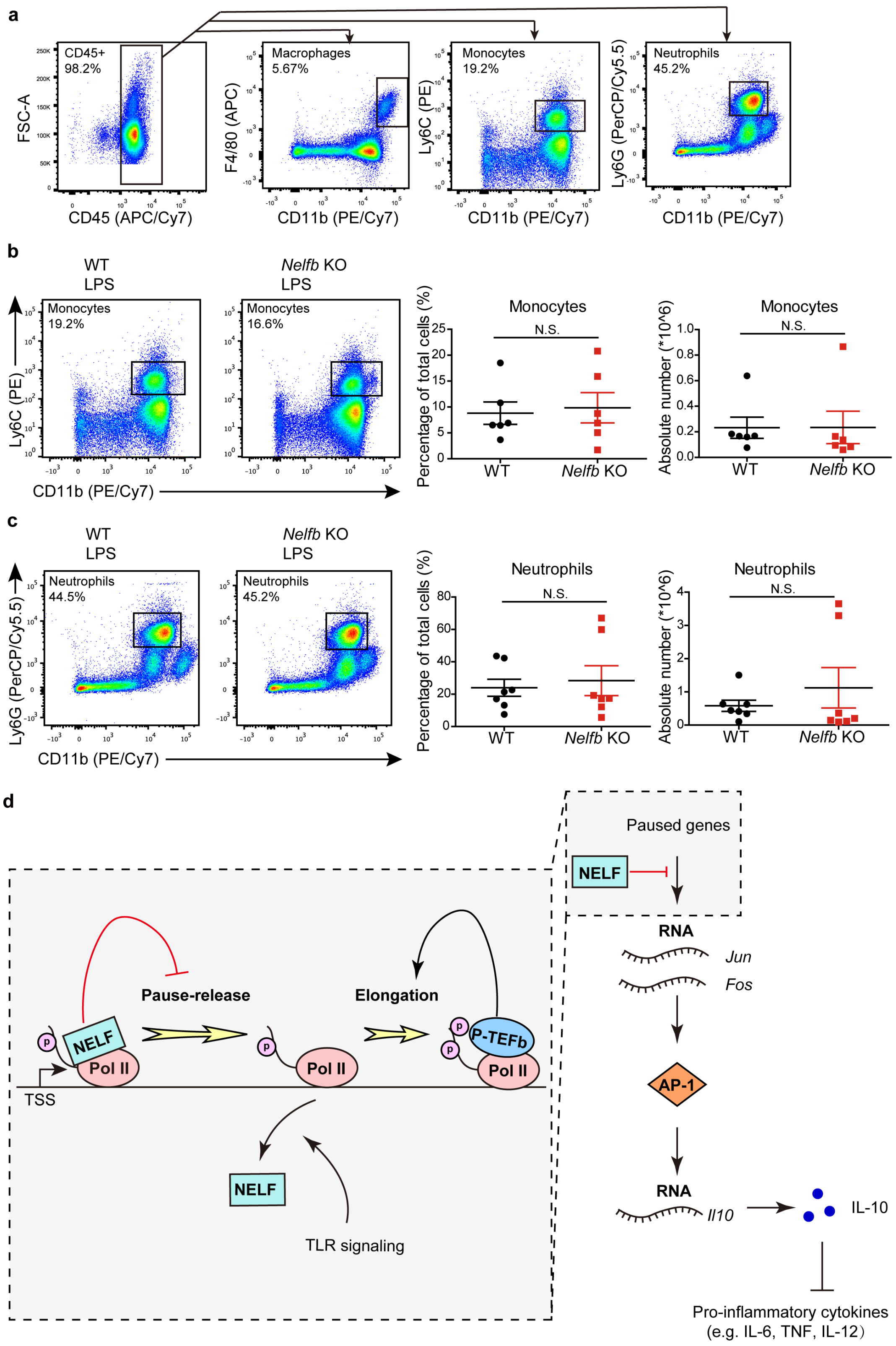

a
CD45+
98.2%
FSC-A
CD45 (APC/Cy7)
Macrophages
5.67%
F4/80 (APC)
CD11b (PE/Cy7)
Monocytes
19.2%
Ly6C (PE)
CD11b (PE/Cy7)
Neutrophils
45.2%
Ly6G (PerCP/Cy5.5)
CD11b (PE/Cy7)
b
WT
LPS
Nelfb KO
LPS
Monocytes
19.2%
Monocytes
16.6%
Ly6C (PE)
CD11b (PE/Cy7)
Monocytes
Percentage of total cells (%)
N.S.
WT
Nelfb KO
Monocytes
Absolute number (*10^6)
N.S.
WT
Nelfb KO
c
WT
LPS
Nelfb KO
LPS
Neutrophils
44.5%
Neutrophils
45.2%
Ly6G (PerCP/Cy5.5)
CD11b (PE/Cy7)
Neutrophils
Percentage of total cells (%)
N.S.
WT
Nelfb KO
Neutrophils
Absolute number (*10^6)
N.S.
WT
Nelfb KO
d
Paused genes
NELF
RNA
Jun
Fos
AP-1
RNA
Il10
IL-10
Pro-inflammatory cytokines
(e.g. IL-6, TNF, IL-12）
Pause-release
Elongation
NELF
Pol II
P-TEFb
p
TSS
NELF
TLR signaling

Supplementary Figure 7. The role of NELF in peritonitis model *in vivo* and working model. (**a**) Gating strategy for FACS analysis of peritoneal cells. $CD45^+$ cells were gated from the total peritoneal cells, and macrophages ($F4/80^+CD11b^+$), monocytes ($CD11b^+Ly6C^+$) and neutrophils ($CD11b^+Ly6G^+$) were subsequently gated from $CD45^+$ cells. (**b**, **c**) FACS analysis of the monocyte (**b**: $CD45^+CD11b^+Ly6C^+$) and neutrophil (**c**: $CD45^+CD11b^+$ $Ly6G^+$) populations in peritoneal exudates of WT and *Nelfb* KO mice 9 h after an intraperitoneal injection of LPS (100 ng/mouse). Representative FACS distribution (left) and cumulative (monocytes: n=6; neutrophils: n=7 biologically independent animals) percentage (middle) as well as absolute cell numbers (right) are shown. N.S. $P>0.05$ by two-sided unpaired Student's *t* test. Error bars indicate SEM. (**d**) Working model: NELF-mediated post-initiation control of the pro- and anti-inflammatory regulation network. Left: Promoter-proximal Pol II pausing landscape in resting macrophages is broadly marked by NELF occupancy. LPS stimulation leads to CDK9-independent NELF dismissal from TSS regions and pause-release. P-TEFb is recruited to phosphorylate Pol II leading to transcription elongation in gene body regions. Right: NELF enforces the transcriptional pause on its direct targets, *Jun* and *Fos*, which, in turn, constrains *Il10* transcription thereby serving a permissive role in pro-inflammatory cytokine production and macrophage-mediated inflammation. Source data are provided as a Source Data file.
