# Supplementary material for: Negative elongation factor complex enables macrophage inflammatory responses by controlling anti-inflammatory gene expression
Source: Nat Commun. 2020 May 8;11:2286. doi: 10.1038/s41467-020-16209-5 (PMC7210294; doi:10.1038/s41467-020-16209-5)
Supplement: Supplementary file 3 — Description of Additional Supplementary Information [file 41467_2020_16209_MOESM3_ESM.pdf]

Description of Additional Supplementary Files

File Name: Supplementary Data 1
Description: Primers and oligonucleotides used in this study.
