# Supplementary material for: Negative elongation factor complex enables macrophage inflammatory responses by controlling anti-inflammatory gene expression
Source: Nat Commun. 2020 May 8;11:2286. doi: 10.1038/s41467-020-16209-5 (PMC7210294; doi:10.1038/s41467-020-16209-5)
Supplement: Supplementary file 5 — Reporting Summary [file 41467_2020_16209_MOESM5_ESM.pdf]

nature research

Corresponding author(s): Xiaoyu Hu, Inez Rogatsky

Last updated by author(s): Mar 21, 2020

Reporting Summary

Nature Research wishes to improve the reproducibility of the work that we publish. This form provides structure for consistency and transparency in reporting. For further information on Nature Research policies, see Authors & Referees and the Editorial Policy Checklist .

Statistics

For all statistical analyses, confirm that the following items are present in the figure legend, table legend, main text, or Methods section.

n/a	Confirmed	
☐	☒	The exact sample size (*n*) for each experimental group/condition, given as a discrete number and unit of measurement
☐	☒	A statement on whether measurements were taken from distinct samples or whether the same sample was measured repeatedly
☐	☒	The statistical test(s) used AND whether they are one- or two-sided<br>*Only common tests should be described solely by name; describe more complex techniques in the Methods section.*
☐	☒	A description of all covariates tested
☐	☒	A description of any assumptions or corrections, such as tests of normality and adjustment for multiple comparisons
☐	☒	A full description of the statistical parameters including central tendency (e.g. means) or other basic estimates (e.g. regression coefficient) AND variation (e.g. standard deviation) or associated estimates of uncertainty (e.g. confidence intervals)
☐	☒	For null hypothesis testing, the test statistic (e.g. *F*, *t*, *r*) with confidence intervals, effect sizes, degrees of freedom and *P* value noted<br>*Give P values as exact values whenever suitable.*
☒	☐	For Bayesian analysis, information on the choice of priors and Markov chain Monte Carlo settings
☒	☐	For hierarchical and complex designs, identification of the appropriate level for tests and full reporting of outcomes
☒	☐	Estimates of effect sizes (e.g. Cohen's *d*, Pearson's *r*), indicating how they were calculated

Our web collection on statistics for biologists contains articles on many of the points above.

Software and code

Policy information about availability of computer code

Data collection	qPCR data were collected by ABI ViiATM 7 Real-Time System (Life Technologies, Thermo Fisher Scientific). FACS data were collected by FACSFortessa flow cytometer (BD Biosciences). Pol II, CDK9 and NELF-E ChIP-seq data and RNA-seq data were collected by HiSeq2500 (Illumina). PU.1 ChIP-seq data were downloaded from NCBI GEO DataSet. PRO-seq data were collected by HiSeq X-ten (Illumina). Detailed information was listed in Methods.
Data analysis	FACS data were analyzed by Flowjo vX 0.7 software (BD Biosciences). GraphPad Prism 5 and R (3.3.0) were used for all graphing and statistical tests as indicated in Methods. Relative density of blotting bands was quantified using Image J (v1.52a) Bowtie (v1.1.2) was used to map the ChIP-seq and PRO-seq data to reference genome, and Tophat (v2.1.0) was used to map the RNA-seq data to reference genome. HOMER software package (v4.7.2) was used to visualize and quantify the ChIP-seq and PRO-seq signal. Cufflinks (v2.2.1) was used to analyze differentially expressed genes from RNA-seq data set. DiffBind (v2.12.0) was used to assess differential NELF occupancy at TSS regions.

For manuscripts utilizing custom algorithms or software that are central to the research but not yet described in published literature, software must be made available to editors/reviewers. We strongly encourage code deposition in a community repository (e.g. GitHub). See the Nature Research guidelines for submitting code & software for further information.

Data

Policy information about availability of data

All manuscripts must include a data availability statement. This statement should provide the following information, where applicable:

- Accession codes, unique identifiers, or web links for publicly available datasets
- A list of figures that have associated raw data
- A description of any restrictions on data availability

RNA-seq, NELF-E ChIP-seq, CDK9 ChIP-seq, Pol II ChIP-seq and PRO-seq data are deposited in the Genome Expression Omnibus under accession numbers GSE122292, GSE123557, GSE122300, GSE103795 and GSE123370, respectively. All data in this manuscript are available upon publication.

Field-specific reporting

Please select the one below that is the best fit for your research. If you are not sure, read the appropriate sections before making your selection.

- [x] Life sciences
- [ ] Behavioural & social sciences
- [ ] Ecological, evolutionary & environmental sciences

For a reference copy of the document with all sections, see nature.com/documents/nr-reporting-summary-flat.pdf

Life sciences study design

All studies must disclose on these points even when the disclosure is negative.

Sample size	No sample size calculations were performed. To ensure reproducibility, we used at least three biological replicates for in vitro experiments. Means of independent experiments or representative data were shown in figures. For in vivo experiments, the numbers of experimental animals used are listed in the figure legends.
Data exclusions	No data exclusions were applied.
Replication	All of the experiments, expecting ChIP-seq and PRO-seq, have been repeated at least 3 times. The times of replication for each experiment were indicated in figure legend. All attempts at replication were successful.
Randomization	For in vitro assays, cells were randomly assigned to either experimental or control groups. For in vivo assays, WT and KO mice with the same age and sex were randomly assigned to specific treatment groups.
Blinding	Investigators were not blinded during data collection as total blindness was not feasible during the animal handling process.

Reporting for specific materials, systems and methods

We require information from authors about some types of materials, experimental systems and methods used in many studies. Here, indicate whether each material, system or method listed is relevant to your study. If you are not sure if a list item applies to your research, read the appropriate section before selecting a response.

Materials & experimental systems

n/a	Involved in the study	
	x	Antibodies
	x	Eukaryotic cell lines
x		Palaeontology
	x	Animals and other organisms
x		Human research participants
x		Clinical data

Methods

n/a	Involved in the study	
	x	ChIP-seq
	x	Flow cytometry
x		MRI-based neuroimaging

Antibodies

Antibodies used	Anti-p38a(C-20)(sc-535), anti-PU.1(T-21)(sc-352), anti-Pol II(sc-9001x), anti-CDK9(H169)(sc-8338), anti-NELF-E(F9)(sc-377052) antibodies were from Santa Cruz Biotechnology. Anti-NELF-B(ab167401) and anti-RNA polymerase II CTD repeat YSPTSPS (phospho S2)(ab5095) antibodies were from Abcam. Anti-NELF-E antibody from Proteintech (10705-1-AP) was also used as indicated. Anti-Ly-6G(560602), anti-Ly6C(560592) and anti-Siglec F(562681) were from BD Bioscience. Anti-CD11b(101216) and anti-CD45R(103116) were from BioLegend. Anti-F4/80(17-4801) and anti-CD11c(25-0114-82) were from eBioscience. Anti-c-Fos (9F6)(2250), anti-c-Jun(60A8)(9165), anti-phospho-p38 MAPK(Thr180/Tyr182)(9215), anti-phospho-NF-κB p65(Ser536)(93H1) (3033), anti-NF-κB p65(C22B4)(4764), anti-phospho-CREB(Ser133)(87G3)(9198), anti-phospho-p44/42 MAPK(Erk1/2)(Thr202/Tyr204)(9101), anti-p44/42 MAPK(Erk1/2)(9102), anti-phospho-SAPK/JNK(Thr183/Tyr185)(9251), anti-SAPK/JNK(9252) antibodies were from Cell Signaling Technology. Anti-mouse IL-10R(CD210)(BE0050) was from BioXCell, and anti-actin(AC026) was from ABclonal.
Validation	No test data was added to the manuscript. Validation information for each antibody can be found in manufacturer's website as listed below.<br>Anti-p38a(C-20)(sc-535), https://www.scbt.com/p/p38alpha-antibody-c-20<br>Anti-PU.1(T-21)(sc-352), https://www.scbt.com/zh/p/pu-1-antibody-t-21<br>Anti-Pol II(sc-9001x), https://www.scbt.com/zh/p/pol-ii-antibody-h-224<br>Anti-CDK9(H169)(sc-8338), https://www.scbt.com/zh/p/cdk9-antibody-h-169<br>Anti-NELF-E(F9)(sc-377052), https://www.scbt.com/zh/p/nelf-e-antibody-f-9<br>Anti-NELF-B(ab167401), https://www.abcam.com/nelf-b-antibody-epr11200-ab167401.html<br>Anti-RNA polymerase II CTD repeat YSPTSPS (phospho S2)(ab5095), https://www.abcam.com/rna-polymerase-ii-ctd-repeat-ysptsps-phospho-s2-antibody-chip-grade-ab5095.html

Anti-NELF-E (Proteintech, 10705-1-AP), https://www.ptglab.com/Products/RDBP-Antibody-10705-1-AP.htm
Anti-Ly-6G(560602), https://www.bdbiosciences.com/cn/reagents/research/antibodies-buffers/immunology-reagents/anti-mouse-antibodies/cell-surface-antigens/percp-cy55-rat-anti-mouse-ly-6g-1a8/p/560602
Anti-Ly6C(560592), https://www.bdbiosciences.com/cn/reagents/research/antibodies-buffers/immunology-reagents/anti-mouse-antibodies/cell-surface-antigens/pe-rat-anti-mouse-ly-6c-al-21/p/560592
Anti-Siglec F(562681), https://www.bdbiosciences.com/cn/applications/research/b-cell-research/surface-markers/mouse/bv421-rat-anti-mouse-siglec-f-e50-2440/p/562681
Anti-CD11b(101216), https://www.biolegend.com/en-us/products/pe-cy7-anti-mouse-human-cd11b-antibody-1921
Anti-CD45R(103116), https://www.biolegend.com/en-us/products/apc-cyanine7-anti-mouse-cd45-antibody-2530
Anti-F4/80(17-4801), https://www.thermofisher.com/cn/zh/antibody/product/F4-80-Antibody-clone-BM8-Monoclonal/17-4801-80
Anti-CD11c(25-0114-82), https://www.thermofisher.com/cn/zh/antibody/product/CD11c-Antibody-clone-N418-Monoclonal/25-0114-82
Anti-c-Fos(9F6)(2250), https://www.cst-c.com.cn/products/primary-antibodies/c-fos-9f6-rabbit-mab/2250
Anti-c-Jun(60A8)(9165), https://www.cst-c.com.cn/products/primary-antibodies/c-jun-60a8-rabbit-mab/9165
Anti-phospho-p38 MAPK(Thr180/Tyr182)(9215), https://www.cst-c.com.cn/products/primary-antibodies/phospho-p38-mapk-thr180-tyr182-3d7-rabbit-mab/9215
Anti-phospho-NF-κB p65(Ser536)(93H1)(3033), https://www.cst-c.com.cn/products/primary-antibodies/phospho-nf-kb-p65-ser536-93h1-rabbit-mab/3033
Anti-NF-κB p65(C22B4)(4764), https://www.cst-c.com.cn/products/primary-antibodies/nf-kb-p65-c22b4-rabbit-mab/4764
Anti-phospho-CREB(Ser133)(87G3)(9198), https://www.cst-c.com.cn/products/primary-antibodies/phospho-creb-ser133-87g3-rabbit-mab/9198
Anti-phospho-p44/42 MAPK(Erk1/2)(Thr202/Tyr204)(9101), https://www.cst-c.com.cn/products/primary-antibodies/phospho-p44-42-mapk-erk1-2-thr202-tyr204-antibody/9101
Anti-p44/42 MAPK(Erk1/2)(9102), https://www.cst-c.com.cn/products/primary-antibodies/p44-42-mapk-erk1-2-antibody/9102
Anti-phospho-SAPK/JNK(Thr183/Tyr185)(9251), https://www.cst-c.com.cn/products/primary-antibodies/phospho-sapk-jnk-thr183-tyr185-antibody/9251
Anti-SAPK/JNK(9252), https://www.cst-c.com.cn/products/primary-antibodies/sapk-jnk-antibody/9252
Anti-mouse IL-10R(CD210)(BE0050) BioXCell, https://bxcell.com/product/m-il-10r/
Anti-actin(AC026) ABclonal, https://abclonal.com.cn/catalog/AC026

Eukaryotic cell lines

Policy information about cell lines

Cell line source(s)	Immortalized bone marrow-derived macrophages (iBMDM) were generated from WT C57BL/6J mice.
Authentication	This cell line was generated in house and was not authenticated.
Mycoplasma contamination	Cell lines were tested negative for mycoplasma contamination.
Commonly misidentified lines (See ICLAC register)	No commonly misidentified lines were used in this study.

Animals and other organisms

Policy information about studies involving animals; ARRIVE guidelines recommended for reporting animal research

Laboratory animals	Nelfb flox/flox mice were generated by Dr. Rong Li's laboratory. Myd88-/- and Lyz2-Cre mice were obtained from Jackson Laboratory. Mice were housed in individually ventilated cages in a temperature and light regulated room (20-26°C, humidity 40-70% and dark/light=12/12) in a SPF facility and received food and water ad libitum in Laboratory Animal Research Center of Tsinghua University.
Wild animals	This study did not involve wild animals.
Field-collected samples	This study did not involve samples collected from fields.
Ethics oversight	The experiments using mice were approved by the Institutional Animal Care and Use Committee at Tsinghua University.

Note that full information on the approval of the study protocol must also be provided in the manuscript.

ChIP-seq

Data deposition

- [x] Confirm that both raw and final processed data have been deposited in a public database such as GEO.
- [ ] Confirm that you have deposited or provided access to graph files (e.g. BED files) for the called peaks.

Data access links
May remain private before publication.

NELF-E ChIP-seq https://www.ncbi.nlm.nih.gov/geo/query/acc.cgi?acc=GSE123557 and the password is qrcfmuuutrgtfkv
CDK9 ChIP-seq https://www.ncbi.nlm.nih.gov/geo/query/acc.cgi?acc=GSE122300 and the password is ijypiyyazbexjqz
PolII ChIP-seq https://www.ncbi.nlm.nih.gov/geo/query/acc.cgi?acc=GSE103795 and the password is idgfsuywzzwplcr
PRO-seq https://www.ncbi.nlm.nih.gov/geo/query/acc.cgi?acc=GSE123370 and the password is yhglswoubpalxsr
RNA-seq https://www.ncbi.nlm.nih.gov/geo/query/acc.cgi?acc=GSE122292 and the password is wbategkexpcjpgf

Files in database submission

Files File type
PolII_UT_rep1.tar.gz Fastq
PolII_UT_rep2.tar.gz Fastq
PolII_LPS_rep1.tar.gz Fastq
PolII_LPS_rep2.tar.gz Fastq
PolII_UT_rep1.ucsc.bedGraph.gz bedGraph
PolII_UT_rep2.ucsc.bedGraph.gz bedGraph
PolII_LPS_rep1.ucsc.bedGraph.gz bedGraph
PolII_LPS_rep2.ucsc.bedGraph.gz bedGraph
NELF_Input_S5_L001_R1_001.fastq Fastq
NELF_UT_rep1.fastq Fastq
NELF_UT_rep1_S10_L002_R1_001.fastq Fastq
NELF_L30_rep1.fastq Fastq
NELF_L30_rep1_S9_L002_R1_001.fastq Fastq
NELF_L60_rep1.fastq Fastq
NELF_L60_rep1_S8_L002_R1_001.fastq Fastq
NELF_UT_rep2_S1_L008_R1_001.fastq Fastq
NELF_UT_rep2_S1_run2_L005_R1_001.fastq Fastq
NELF_L30_rep2_S2_L008_R1_001.fastq Fastq
NELF_L30_rep2_run2_S2_L005_R1_001.fastq Fastq
NELF_L60_rep2_S3_L008_R1_001.fastq Fastq
NELF_L60_rep2_run2_S3_L005_R1_001.fastq Fastq
NELF_UT_rep1.ucsc.bedGraph.gz bedGraph
NELF_L30_rep1.ucsc.bedGraph.gz bedGraph
NELF_L60_rep1.ucsc.bedGraph.gz bedGraph
NELF_UT_rep2.ucsc.bedGraph.gz bedGraph
NELF_L30_rep2.ucsc.bedGraph.gz bedGraph
NELF_L60_rep2.ucsc.bedGraph.gz bedGraph
CDK9_UT.fastq.gz Fastq
CDK9_L60.fastq.gz Fastq
CDK9_UT.fastq.gz Fastq
CDK9_L60.fastq.gz Fastq
CDK9_UT.bedGraph.gz bedGraph
CDK9_L60.bedGraph.gz bedGraph

Genome browser session
(e.g. UCSC)

http://genome.ucsc.edu/cgi-bin/hgTracks?db=mm10&lastVirtModeType=default&lastVirtModeExtraState=&virtModeType=default&virtMode=0&nonVirtPosition=&position=chr1%3A3000470-3001063&hgsid=716297353_A3BSATf2E7cNv0CLppD9qSduHWuc

Methodology

Replicates

Pol II ChIPseq (2 biological replicates for each conditions), NELF-E ChIPseq (2 biological replicates for each conditions), CDK9 ChIPseq (one replicate)

Sequencing depth

Samples Total reads Uniquely mapped reads Length
PolII_UT_rep1 91040220 67080518 Single-end 50
PolII_UT_rep2 92349914 65557897 Single-end 50
PolII_LPS_rep1 95355253 69452059 Single-end 50
PolII_LPS_rep2 96114864 66920965 Single-end 50
NELF_Input 74540651 51388956 Single-end 50
NELF_UT_rep1 88685924 58430837 Single-end 50
NELF_L30_rep1 70212340 46730418 Single-end 50
NELF_L60_rep1 83921847 56597077 Single-end 50
NELF_UT_rep2 162422256 96829803 Single-end 50
NELF_L30_rep2 104392003 71356586 Single-end 50
NELF_L60_rep2 121102156 80337260 Single-end 50
CDK9_UT 64164547 40888594 Single-end 50
CDK9_L60 61091204 40346767 Single-end 50

Antibodies

Pol II (sc-9001x, Santa Cruz), NELF-E (F9 sc-377052, Santa Cruz), CDK9 (H-169 sc-8338, Santa Cruz)

Peak calling parameters	ChIP-seq reads in fastq files were aligned to mouse genome (UCSC mm10) using Bowtie (version 1.1.2) to generate alignment files of uniquely mapped reads with maximum allowed mismatch of 2 (-m 1 –n 2) for each data set. Peak calling was applied to NELF-E ChIPseq data set, which was implemented by HOMER with FDR>0.001 to input condition.
Data quality	For Pol II ChIPseq data sets, we tested the correlation for ChIPseq signals every 10kb across the genome between two biological replicates for each conditions, and correlation coefficient (R) for untreated condition is 0.99 and for LPS stimulated condition is 0.85.
Software	Mapping: Bowtie (version 1.1.2); peak calling: findPeaks program in HOMER (v4.7.2)

Flow Cytometry

Plots

Confirm that:

- [x] The axis labels state the marker and fluorochrome used (e.g. CD4-FITC).
- [x] The axis scales are clearly visible. Include numbers along axes only for bottom left plot of group (a 'group' is an analysis of identical markers).
- [x] All plots are contour plots with outliers or pseudocolor plots.
- [x] A numerical value for number of cells or percentage (with statistics) is provided.

Methodology

Sample preparation	Single cell suspensions were made from organs (spleen, lung) or isolated from peritoneal cavity.
Instrument	FACSFortessa flow cytometer (BD Biosciences)
Software	Flowjo software (BD Biosciences)
Cell population abundance	No sorting was performed. For population abundance in FACS analysis experiments, see specific figures for detailed information.
Gating strategy	Cells were gated first on FSC-A versus SSC-A to determine include all viable cells (P1), on FSC-H versus FCS-W to eliminate doublets (P2), and then on APC-Cy7 (CD45) versus FSC-A to specify leukocytes (P3). The following gating strategies are antibodyspecific for determination of specific immune cell populations.

- [x] Tick this box to confirm that a figure exemplifying the gating strategy is provided in the Supplementary Information.
